# Macular Dystrophy with Bilateral Macular Telangiectasia Related to the CYP2U1 Pathogenic Variant Assessed with Multimodal Imaging Including OCT-Angiography

**DOI:** 10.3390/genes12111795

**Published:** 2021-11-15

**Authors:** Khaled El Matri, Yousra Falfoul, Imen Habibi, Ahmed Chebil, Daniel Schorderet, Leila El Matri

**Affiliations:** 1 Department B, Institut Hédi Rais D’ophtalmologie de Tunis, Tunis 1007, Tunisia; khaled.elmatri@gmail.com (K.E.M.); chebilahmed@yahoo.fr (A.C.); leilaelmatri@gmail.com (L.E.M.); 2 Oculogenetic Laboratory LR14SP01, Institut Hédi Rais D’ophtalmologie de Tunis, Tunis 1007, Tunisia; 3 El Manar/Faculté de Médecine de Tunis, Université de Tunis, Tunis 1007, Tunisia; 4 IRO—Institut de Recherche en Ophtalmologie, 1950 Sion, Switzerland; habibiimen@hotmail.com (I.H.); daniel.schorderet@irovision.ch (D.S.); 5 Faculté de Biologie et de Médecine, Université de Lausanne, 1005 Lausanne, Switzerland; 6 Faculté des Sciences de la Vie, École Polytechnique Fédérale de Lausanne, 1015 Lausanne, Switzerland

**Keywords:** *CYP2U1*, SPG56, macular telangiectasia, choroidal neovascularization, OCT-Angiography, multimodal imaging

## Abstract

Purpose: We report the case of a neurologically asymptomatic young boy presenting with an unusual phenotype of *CYP2U1* related macular dystrophy associating bilateral macular telangiectasia (MacTel) and fibrotic choroidal neovascularization (CNV), assessed with complete multimodal imaging including optical coherence tomography angiography (OCT-A). Case presentation: A twelve-year-old boy from a non-consanguineous family complained of bilateral progressive visual loss and photophobia. The best-corrected visual acuity was 2/10 on the right eye and 3/10 on the left eye. Fundus examination showed central pigmented fibrotic macular scar and yellowish punctuate deposits in both eyes. En face OCT-A detected typical macular telangiectasia (MacTel) in both eyes with dilated telangiectatic capillaries in the deep capillary plexus associated with vascular anomalies in the superficial and deep capillary plexus. Typical hypo-reflective cavities were observed within the inner foveal layers on structural OCT. En face OCT-A also confirmed the presence of bilateral inactive CNV within the fibrotic scars, showing high-flow vascular network at the level of the subretinal hyperreflective lesions. Whole exome sequencing identified a known homozygous pathogenic variant in *CYP2U1* gene (c.1168C > T, p.Arg390*), which is a disease-causing mutation in autosomal recessive spastic paraplegia type 56 (SPG56). The neurological examination was normal, and electromyography and brain magnetic resonance imaging were unremarkable as well. Conclusion: Macular dystrophy can be the first manifestation in SPG56. A particular phenotype with MacTel was observed, and neovascular complications are possible. *CYP2U1* should be included in the panels of genes tested for macular dystrophies, especially in the presence of MacTel and/or neurological manifestations.

## 1. Introduction

*CYP2U1* is a human thymus and brain-specific cytochrome P450, playing an important role in the fatty-acid metabolism, such as docosahexaenoic acid (DHA) and eicosapentaenoic acid (EPA), which are found in high concentrations in the brain and retina [1]. Pathogenic variants in *CYP2U1* gene are disease-causing mutations in autosomal recessive spastic paraplegia type 56 (SPG56) [2,3,4]. Very few cases of macular dystrophies related to *CYP2U1* pathogenic variants have been reported in the literature [4,5,6].

We report a case of a young boy presenting with bilateral macular telangiectasia (MacTel) and fibrotic choroidal neovascularization (CNV) related to pathogenic variant in *CYP2U1*. We present clinical and complete multimodal imaging of the case, including optical coherence tomography angiography (OCT-A).

## 2. Case Presentation

A twelve-year-old boy with no medical history nor known ocular history complained of bilateral progressive visual loss and severe photophobia. There was no consanguinity reported no similar cases in the family. His best-corrected visual acuity was 2/10 in the right eye (RE) and 3/10 in the left eye (LE).

**Figure 1:**
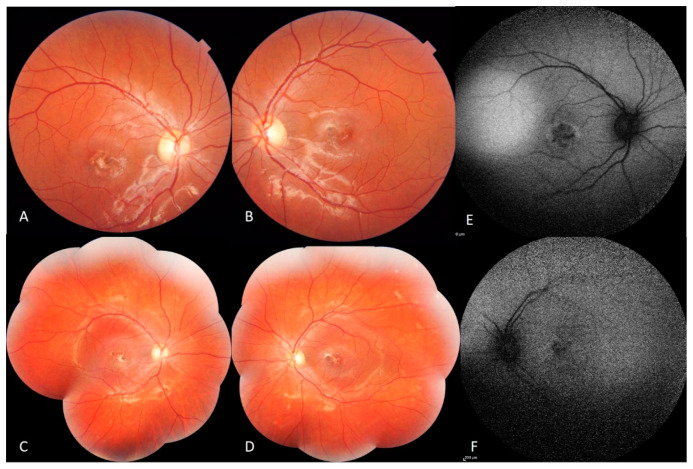
Color fundus photography (CFP) and blue-light FAF of macular dystrophy in the RE. (**A**,**B**) CFP: Macular fibrotic-pigmented lesion associated with yellowish punctuate deposits. Normal appearance of optic disc. (**C**,**D**) normal peripheral retina. (**E**,**F**) Blue-light FAF: Heterogeneous patchy central autofluorescence with increased perifoveal autofluorescence.

Fundus examination showed macular fibrotic-pigmented lesion associated with yellowish punctuate deposits in both eyes with heterogeneous patchy central autofluorescence and increased perifoveal autofluorescence on blue fundus autofluorescence (FAF) (Heidelberg, HRA2, Heidelberg, Germany). The peripheral retinal examination was normal. The optic disc appearance was normal in both eyes (Figure 1).

**Figure 2:**
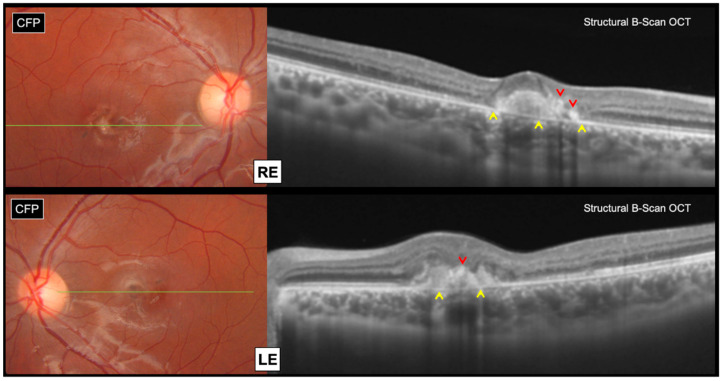
Swept source structural B-Scan OCT of macular lesions in both eyes (green lines on CFP corresponding to macular OCT scan levels). Bilateral subretinal hyperreflective lesions, without any subretinal fluid accumulation. Focal areas of outer retinal atrophy with choroidal hyper-transmission (yellow arrowheads). Intraretinal pigment migration with back-shadowing (red arrowheads).

Swept source structural optical coherence tomography (OCT) (Topcon, DRI OCT Triton, Tokyo, Japan) revealed bilateral subretinal hyperreflective lesions, associated with focal areas of outer retinal atrophy and intraretinal pigment migration, without any subretinal fluid accumulation (Figure 2).

**Figure 3:**
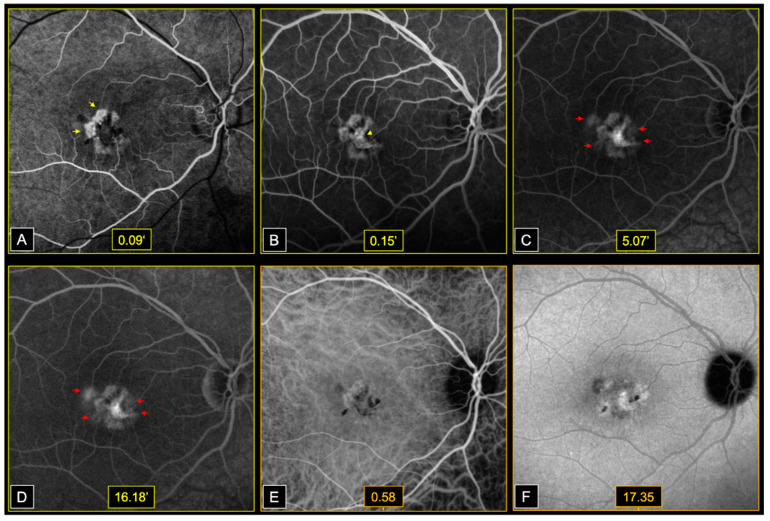
Dye angiography of macular fibrotic lesion and perifoveal telangiectasia in the RE. (**A**,**B**)—Early phases FA: Window defects at the level of areas of atrophy (yellow arrows—**A**), followed by dye filling of the central fibrotic lesion (yellow arrowheads—**B**). (**C**,**D**)—Late phases FA: Perifoveal capillary dye leakage (red arrows). (**E**,**F**)—Early and late phase ICGA: Speckled appearance of the macula. Heterogenous areas of hypo and hyperfluorescence.

Early phases fluorescein angiography (FA) (Heidelberg, HRA2, Heidelberg, Germany) showed window defects at the level of areas of atrophy, followed by dye filling of the central fibrotic lesions, in both eyes. Bilateral perifoveal capillary dye leakage was observed in late FA phases (Figure 3).

On infracyanine green angiography (ICGA) (Heidelberg, HRA2, Heidelberg, Germany), we noted a speckled appearance of the macula with heterogenous areas of hypo and hyperfluorescence, in both eyes (Figure 3).

**Figure 4:**
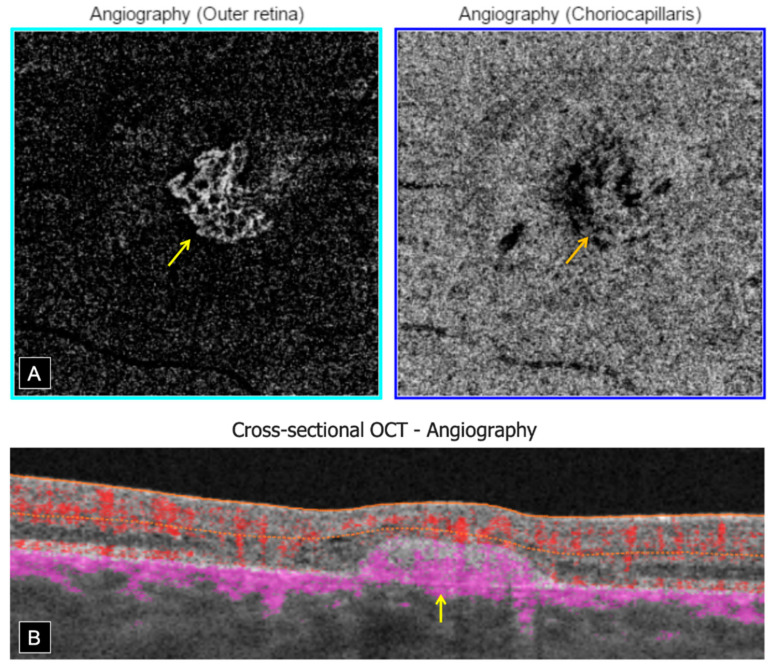
Swept source OCT-Angiography of CNV in the RE. (**A**)—En face OCT-A (3 × 3 mm): Well-defined high-flow vascular network at the level of the OR slab (yellow arrow) with a projection in the CC slab (orange arrow). (**B**)—Cross-sectional OCT-A: High-flow signal within subretinal hyper-reflective lesion (yellow arrow).

Swept source OCT-A (Topcon, DRI OCT Triton, Tokyo, Japan) confirmed the presence of bilateral inactive macular CNV within the fibrotic scars, showing a well-defined high-flow vascular network on en face imaging, at the level of the outer retina (OR) slab with a projection in the choriocapillaris (CC) slab. Cross-sectional OCT-A showed a high-flow signal within subretinal hyper-reflective lesions (Figure 4).

**Figure 5:**
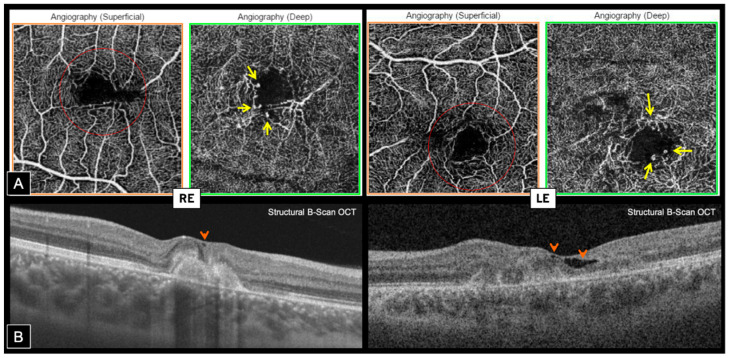
Swept source OCT and OCT-Angiography of macular telangiectasia in both eyes. (**A**)—En face OCT-A (3 × 3 mm): Perifoveal tortuous capillaries and vascular voids in the SCP (red circle). Perifoveal markedly dilated telangiectatic and rarified capillaries in the DCP (yellow arrows). Distorted FAZ in both SCP and DCP. Vascular anomalies are mainly temporal in the RE and more circumferential in the LE. (**B**)—Structural B-Scan OCT: Hyporeflective cystic cavities within inner foveal layers (orange arrowheads), around areas of vascular anomalies.

En face OCT-A highlighted the presence of typical MacTel in both eyes, revealing perifoveal markedly dilated telangiectatic and rarified capillaries in the deep capillary plexus (DCP) associated with perifoveal tortuous capillaries and vascular voids in the superficial capillary plexus (SCP). Foveal avascular zone (FAZ) was distorted in both SCP and DCP, and vascular anomalies were mainly temporal on the RE and more circumferential on the LE (Figure 5A). Hyporeflective cystic cavities were noted on structural OCT within the inner foveal layers, around areas of vascular anomalies, which is consistent with MacTel (Figure 5B).

**Figure 6:**
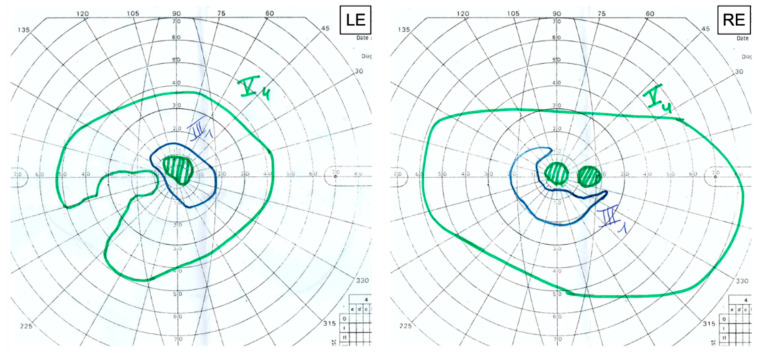
GVF defects of both eyes. Reduced isopters (V4 and III) and central scotomas (V4) in both eyes. Enlarged blind spot (V4) and temporal superior defect excluding the blind spot and the central field (III1) in the RE. Exclusion of the blind spot (V4) in the LE.

Goldmann visual field (GVF) revealed reduced isopters associated with central scotomas in both eyes. We noted an enlarged blind spot associated with a temporal superior defect with exclusion of the blind spot and the central field in the RE and an exclusion of the blind spot in the LE (Figure 6).

**Figure 7:**
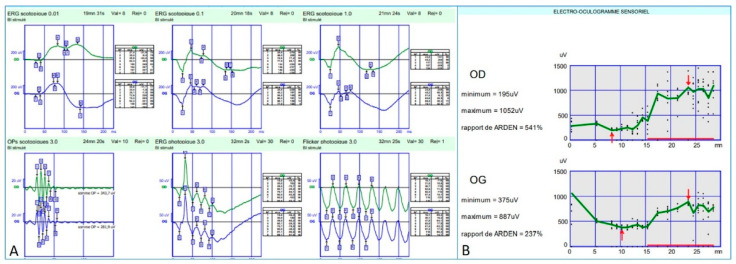
Electrophysiology findings in both eyes. (**A**) f-ERG: Rod response amplitude and peak time within normal limits in scotopic conditions. Normal oscillatory potentials (preserved macular function). Photopic responses amplitude and peak time within normal limits. Regular periodic flicker responses (preserved M- and L-cones function). (**B**)- EOG: Arden ratio within normal limits.

Full-field electroretinogram (ff-ERG) (Metrovision, Perenchies, France) was within normal limits in both scotopic and photopic conditions. Indeed, the rod response amplitude and peak time were within the normal limits in scotopic conditions; oscillatory potentials were normal indicating a preserved macular function; photopic response amplitude and peak time were within normal limits as well; and finally, a flicker ERG showed regular periodic responses reflecting preserved M- and L-cones function (Figure 7A).

Electro-oculogram (EOG) (Metrovision, Perenchies, France) revealed an Arden ratio within normal limits in both eyes (Figure 7B).

In the presence of macular dystrophy, we asked for genetic analysis starting with BEST1 and PRPH2 genes sequencing. However, no pathogenic variant was found in either gene.

We completed our assessment with a whole exome sequencing (WES) that identified a known homozygous pathogenic variant in CYP2U1 gene (c.1168C > T, p.Arg390*), which is a disease-causing mutation in SPG56 [5]. Bioinformatic analysis of the reads containing the c.1168C > T variant did not show any CNV, and it is likely that the patient is homozygous for the pathogenic variant (c.1168C > T, p.Arg390* pathogenic variant). However, compound heterozygous variants, including the c.1168C > T together with a deletion of exon3 cannot be excluded.

**Figure 8:**
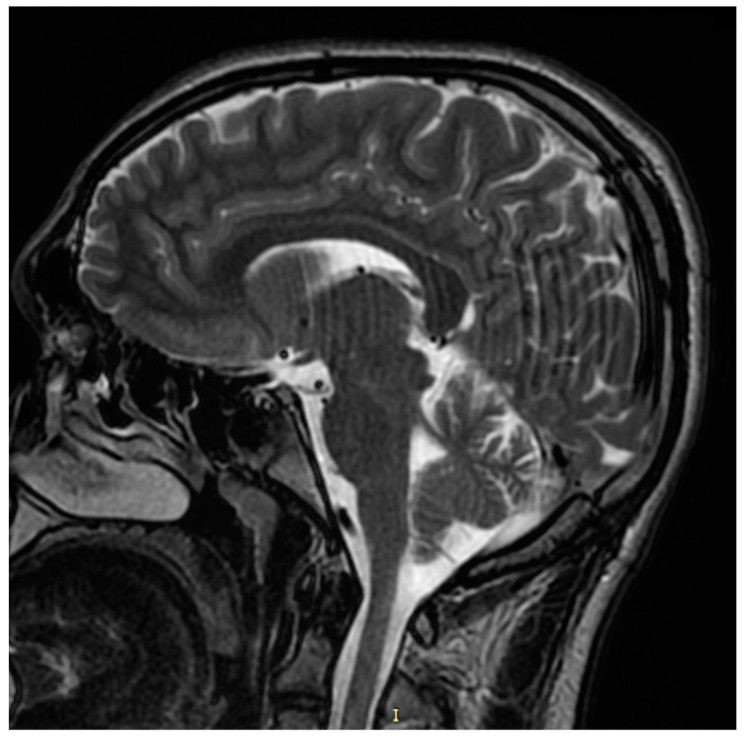
Brain MRI—Sagittal T2-weighted section. Normal brain imaging.

Our patient was neurologically asymptomatic; however, anamnesis revealed a history of walking and speech delay according to his parents. The specialized neurological clinical examination was unremarkable, electromyography (EMG) was normal, and magnetic resonance imaging (MRI) of the brain and spine was unremarkable as well (Figure 8). On optic disc OCT, the peripapillary retinal nerve fiber layer (RNFL) thickness was within normal limits in both eyes.

Whole exome capture was performed on the index patient using the Roche NimbleGen version 2 (44.1–megabase pair) paired-end sample preparation kit and Illumina HiSeq2000 (Illumina, San Diego, CA, USA) at a mean coverage ×31 by Otogenetics Corporation (Norcross, GA, USA). Sequence reads were aligned to the human genome reference sequence (build hg19), and variants were identified after many steps of filtering (Habibi I., 2020). The pathogenicity index for the identified missense mutations was calculated in silico using ClinVar (https://www.ncbi.nlm.nih.gov/clinvar/ (accessed on 5 October 2021). Pathogenic variants were confirmed by Sanger sequencing; however, segregation analysis was not done for the family.

Comprehensive ophthalmic examination and ocular assessment was performed in both parents and was strictly normal.

## 3. Discussion

We report the case of a young boy patient presenting an unusual macular dystrophy related to *CYP2U1* pathogenic variant (c.1168C > T, p.Arg390*) associating bilateral fibrotic CNV, punctiform yellowish deposits, and perifoveal telangiectasia. OCT-A confirmed the neovascular complication, showing a well-defined vascular network in the outer avascular retina at the level of the fibrotic scars. Interestingly, OCT-A also highlighted the presence of typical perifoveal telangiectasia in both eyes with dilated telangiectatic capillaries in the DCP associated with distorted FAZ in both SCP and DCP with tortuous and rarified capillaries. 

Hypo-reflective cystic cavities were present within the inner foveal layers on the corresponding structural B-Scan OCT, and we observed perifoveal capillary dye leakage of FA. In the presence of macular dystrophy with yellowish deposits and fibrotic CNV, the diagnosis was initially guided to a particular phenotype of vitelliform macular dystrophy. We, therefore, asked for genetic analysis, staring with *BEST1* and *PRPH2* gene sequencing. However, no pathogenic variant was found in either gene. Finally, we identified a known homozygous pathogenic variant in *CYP2U1* (c.1168C > T, p.Arg390*), which is a disease-causing mutation in SPG56 [5].

*CYP2U1* is a human thymus and brain-specific cytochrome P450, playing an important role in the fatty-acid metabolism, for instance DHA and EPA, which are found in high concentrations in the brain and retina [1]. Hereditary spastic paraplegia (HSP) is one of most clinically and genetically heterogenous group of neurological diseases, with mutations identified in 22 genes, and at least 48 loci have been mapped accounting for all classical modes of inheritance [7,8].

SPG 56 is an autosomal recessive form of HSP that has a material basis in a mutation in the *CYP2U1* gene on chromosome 4q25. Tesson et al. demonstrated that SPG 56 pathophysiology included alteration of mitochondrial bioenergetic function [4]. It is characterized by early-onset slowly progressive lower-limb weakness and spasticity resulting in walking difficulties. The upper limbs can also be affected, and subclinical axonal neuropathy may be present in some patients [4]. Complicated forms of SPG56 can include additional neurological signs, such spastic quadriparesis, amyotrophy, cerebral or cerebellar atrophy, optic atrophy, and peripheral neuropathy, and/or extra-neurological signs [4].

The largest series of SPG56 related to *CYP2U1* mutations was reported in 2012 by Tesson et al. [4]. There were 11 affected patients from five families, with only one patient presenting with an unspecified macular dystrophy. This macular dystrophy was shortly cited without any thorough structural, vascular, or functional assessment. Then, in 2016, a pigmentary degenerative maculopathy was reported by Leonardi et al. as the prominent phenotype in three members of a consanguineous Italian SPG56 family that harbored a novel homozygous pathogenic variant c.1168C > T, p.Arg390* in *CYP2U1* [5]. 

The same homozygous variant was identified in our patient. All three patients reported in the Leonardi et al. case series had progressive spastic paraplegia associated with other neurological signs [5]. Comprehensive ophthalmologic examination was performed in two patients, revealing bilateral chronic degenerative maculopathy with pigmentary changes and mild temporal sectoral optic disc paleness. Structural OCT showed a disruption of all outer retinal layers and reduced temporal RNFL thickness. Visual evoked potentials abnormalities were observed in both patients. Leonardi et al. did not report the presence of fibrotic CNV within the degenerative maculopathy as observed in our patient, despite the fact that the Amsler grid test denoted metamorphopsia in both eyes of both patients, and they did not perform FA or OCT-A in order to highlight this type of lesion.

Lenoardi et al. did not report the presence of MacTel either [5]; however, as stated before, neither OCT-A nor FA was used to diagnose these vascular anomalies. Structural OCT revealed late-stage macular atrophy. Therefore, indirect structural OCT signs of MacTel, such as hyporeflective cystic cavities, could not be reported. However, the two patients were aged between 42 and 50 at the time of ophthalmic assessment, while ocular signs started between the age 25 and the late 30s in the aforementioned patients. Such structural signs might have been present if OCT had been performed at the beginning of visual complaints.

Our young 12-year-old patient is from a non-consanguineous Tunisian family. Lenoardi et al. did not provide information on the ethnicity of these families, which might suggest a possible common origin with our case [5]. Our patient had a history of walking and speech delay according to his parents, but he was otherwise neurologically asymptomatic at the time of consultation. Our patient’s main complaint was bilateral visual loss and photophobia secondary to his bilateral fibrotic macular dystrophy. The optic disc appearance was normal, and the peripapillary RNFL thickness was within normal limits. 

The neurological clinical examination was unremarkable, and his EMG was normal, excluding SPG56 subclinical axonal neuropathy. The brain MRI was unremarkable as well, excluding SPG56 signs as corpus callosum atrophy, white matter anomalies, cerebellar atrophy, and basal ganglia calcification [4,5]. However, GVF revealed an enlarged and excluded blind spot, which could be a sign of sub-clinical damage of the optic nerve and implies long-term monitoring of the patient.

In the Leonardi et al. case series of SPG56/*CYP2U1* related to the same homozygous pathogenic variant c.1168C > T, p.Arg390* [5], neurological signs started after the age of 30 years in all three patients, while our patient was only 12 years old at the time of macular dystrophy diagnosis. In one case, ocular manifestations started at least 5 years before the onset of motor manifestations. Therefore, a long follow-up is necessary in order to conclude if this pathogenic variant will later be responsible of progressive spastic paraplegia in our patient or if the macular dystrophy will remain the only phenotype.

Recently, a neurologically asymptomatic patient with *CYP2U1*-related maculopathy was reported [6]. He was from a non-consanguineous Swiss family, and his brother was diagnosed with HSP56. Both brothers had compound heterozygous mutations c [452 C > T], p [Pro151Leu]; c [943 C > T], and p [GLN315*] in *CYP2U1.* The authors did not report the presence of perifoveal telangiectasia, and the OCT-A in their case was within normal limits.

To our knowledge, this is the first report of a *CYP2U1*-related maculopathy associating CNV and typical macular telangiectasia. In fact, our findings were similar to the tomographic degenerative structural changes [9] and telangiectatic vascular changes [10] observed in type 2 macular telangiectasia (MacTel2).

In the presence of bilateral fibrotic neovascular macular scar and yellowish deposits associated with perifoveal macular telangiectasia in a twelve-year-old boy, two main diagnoses were discussed. It could be a proliferative stage MacTel2 with fibrotic CNV in a young patient or more likely a hereditary macular dystrophy with neovascular complication, associated with unusual vascular anomalies, such as perifoveal telangiectasia.

In previous studies, approximately 5% of patients with MacTel2 presented foveal yellowish lesions [11,12]. OCT revealed a prominent mound of material between the ellipsoid zone and retinal pigment epithelium (RPE) [12,13]. The material was hyper-autofluorescent and ultra-structurally composed of photoreceptor outer segments debris, similar to that found in macular vitelliform dystrophy with some significant differences [14].

Perifoveal capillary hyper-permeability has also been reported on FA in cases of macular dystrophies, such as adult onset vitelliform macular dystrophy. It was described as characteristic and was thought to be responsible for the pseudo-vitelliform lesions appearing as a result of equilibrium established between a chronic leakage and an active attempt at resorption of accumulating lipid material [15].

OCT-A seems to be an excellent tool for CNV screening in macular dystrophies. In our case, it could detect bilateral CNV within the fibrotic lesions while this was not evident on standard imaging instruments. FA and structural OCT analysis can be difficult in the presence of macular scars due to retinal and RPE changes. Moreover, OCT-A allowed non-invasive investigation of retinal vasculature layer-by-layer and highlighted vascular anomalies that were poorly detected with standard invasive vascular imaging, revealing well-defined perifoveal telangiectatic and rarified capillaries as well as distorted FAZ in both eyes.

Dilated capillaries and perivascular rarefaction have been reported on OCT-A in other macular dystrophies, such as vitelliform dystrophies [16]. It has been hypothesized that vascular rarefaction in both SCP and DCP could play a significant role in the progression of the disease due to reduced blood supply [16,17]. Our case did not show simply dilated capillaries as observed in the Parodi et al. [16] study, but it showed typical MacTel with characteristic structural and vascular changes [9,10].

The perifoveal telangiectatic capillaries observed in our case could be secondary to a chronic ischemic process of the outer retina due to the fibrotic scars and could arise from retrograde neurovascular degeneration as hypothesized by Parodi et al. for the origin of dilated capillaries observed in vitelliform dystrophies [16]. However, the pathophysiology of this perifoveal telangiectasia is more likely to be secondary to similar neurodegenerative mechanisms observed in MacTel2 patients. Indeed, a recent study showed that the lipid metabolism was also implicated in the genesis mechanism of MacTel2 [18]. Elevated levels of deoxsphingolipids were found to be risk factors of MacTel2 as well as for peripheral neuropathy. A different class of lipid than DHA/EPA was implicated here; however, further research studies should be done in this direction to better understand the pathophysiology of both diseases.

Our patient exhibited unprecedently described retinal vascular lesions, allowing us to extend the phenotypic spectrum of *CYP2U1*/SPG56. Larger series of microvascular analysis in CYP2U1-related macular dystrophies using OCT-A are needed to better understand if MacTel is secondary to the neurovascular degenerative process of the disease or if it simply represents a coincident finding.

## 4. Conclusions

In conclusion, macular dystrophy can be observed in SPG56 related to mutations in the *CYP2U1* gene, and this can be its first manifestation. Patients carrying these mutations need to be followed up with in the future to investigate for any late neurological involvement. A particular phenotype with MacTel can be observed and neovascular complications are possible. Finally, *CYP2U1* should be included in the panels of genes tested for macular dystrophies, especially in the presence of MacTel and/or neurological manifestations.

## Data Availability

The data presented in this study are available on request from the corresponding author. The data are not publicly available due to the privacy of patients.
